# Prevalence and Transmission Cycle of Avian Pathogens in the Isolated Oceanic Islands of Japan

**DOI:** 10.1002/ece3.70737

**Published:** 2024-12-23

**Authors:** Mizue Inumaru, Rui Kimura, Naoko Suzuki, Hajime Suzuki, Kazuo Horikoshi, Isao Nishiumi, Kazuto Kawakami, Yoshio Tsuda, Koichi Murata, Yukita Sato

**Affiliations:** ^1^ Department of Medical Entomology National Institute of Infectious Diseases Shinjuku Tokyo Japan; ^2^ Laboratory of Biomedical Science, Department of Veterinary Medicine, College of Bioresource Sciences Nihon University Fujisawa Kanagawa Japan; ^3^ Institute of Boninology, NPO Tokyo Japan; ^4^ Department of Zoology National Museum of Nature and Science Ibaraki Japan; ^5^ Forestry and Forest Products Research Institute Ibaraki Japan; ^6^ Yokohama Zoological Gardens “ZOORASIA” Yokohama Kanagawa Japan; ^7^ Laboratory of Veterinary Parasitology, Department of Veterinary Medicine, Faculty of Agriculture Iwate University Iwate Japan

**Keywords:** APV, avian haemosporidia, Bonin Islands, mosquito, oceanic islands

## Abstract

Avian haemosporidian parasites and avian pox virus (APV) are well‐known pathogens for their impact on avian populations, especially in oceanic islands where introduced pathogens show strong virulence for endemic and naïve birds. The Bonin Islands are a group of oceanic islands 1000 km south of Tokyo. Like the Hawaiian Islands, there are many endemic and endangered species as well as introduced species, which have greatly affected the native avian fauna. However, pathogens in wild birds of this archipelago had not been investigated. In this study, we investigated the prevalence of avian haemosporidian parasites and APV among birds and mosquitoes in this unique ecosystem of the Bonin Islands. From 2014 to 2020, 524 birds of 39 species either rescued, deceased, or caught by mist‐netting were sampled. APV‐like lesions were sampled from nine birds. 262 mosquitoes were collected by sweeping nets or CDC traps. All samples were tested via PCR for haemosporidian infection, and lesions were tested for APV.209 birds (39.9%) of 11 species were positive for haemosporidian parasite DNA, and all three parasite genera were detected. Prevalence was particularly high for *Plasmodium elongatum* (pGRW06) and *Prelictum relictum* (pGRW04). The former was detected from both resident birds and mosquitoes, suggesting local transmission. An introduced species, the warbling white‐eye ( *Zosterops japonicus* ), had a particularly high prevalence of pGRW06 (68.3%) and may be a reservoir of this lineage. Both APV and *Plasmodium* spp. were detected from all APV‐tested birds, suggesting that these two pathogens may be transmitted simultaneously via mosquitoes. The presence of avian haemosporidian parasites and APV was confirmed in the Bonin Islands for the first time. However, the virulence and origin of these pathogens remain unknown, and many bird species are still understudied. Further investigations are required to contribute to the conservation of this unique avifauna.

## Graphical abstract

Avian haemosporidian parasites and avian pox virus were detected from birds of the Bonin Islands of Japan for the first time, including some that are endemic to the islands. Both pathogens are suggested to be simultaneously transmitted by mosquitoes. Some of the haemosporidian parasite lineages are suggested to have been introduced along with their avian hosts.

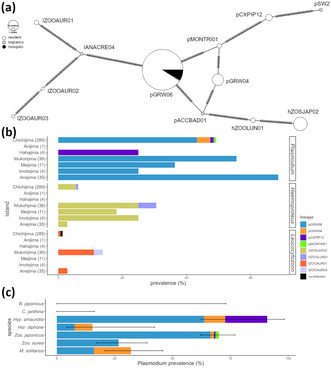

## 1 Introduction

Oceanic islands have isolated history, as they arose from the ocean basin and have therefore never been connected to continental lands. The biotas of the islands are restricted to organisms that were capable of reaching the islands by air or sea, either by passive or active movement. Consequently, unique ecosystems are created through the evolution of endemism (Carlquist 1974; Kawakami and Okochi 2010; Kueffer, Drake, and Fernández‐Palacios 2014). Species of such unique ecosystems are incredibly vulnerable to introduced alien species, which can drastically alter the ecosystem through direct predation, interspecific competition, and the introduction of novel pathogens (Carlquist 1974; Van Riper et al. 1986; Kawakami and Okochi 2010; Kawakami 2019). Avian malaria, caused by *Plasmodium* parasites, and fowlpox caused by avian poxvirus (APV), are known to have caused population‐level lethal impacts on the avifauna of Hawaii and New Zealand (Warner 1968; Van Riper et al. 1986; Van Riper, Van Riper, and Hansen 2002; Van Riper III 1991; Aruch et al. 2007; Atkinson and Lapointe 2009; Howe et al. 2012; Sijbranda et al. 2016; Samuel et al. 2018). Several native honeycreepers of Hawaii were driven into extinction due to the combination of the two pathogens (Warner 1968; Van Riper et al. 1986; Van Riper, Van Riper, and Hansen 2002). Because endemic species on these oceanic islands had never been exposed to these pathogens, they had not developed immunity against these pathogens and were therefore extremely naïve, resulting in high virulence (Van Riper et al. 1986; Atkinson et al. 2000, 2013; Sorci 2013). Introductions of infected avian populations have been considered to be the primary drivers of such population declines and extinctions (Warner 1968; Van Riper III 1991; Van Riper, Van Riper, and Hansen 2002; Ewen et al. 2012). Both avian malaria parasites and APV are known to be transmitted by mosquitoes (Valkiūnas 2005; Aruch et al. 2007; Yeo et al. 2019), although APV can also be transmitted by other mechanical vectors and by direct contact (Huong et al. 2014; Lee et al. 2017; Yeo et al. 2019). Therefore, the introduction of mosquitoes to Hawaii, where suitable vectors were previously absent, incidentally made transmission within the islands possible (Warner 1968; Van Riper et al. 1986; Van Riper, Van Riper, and Hansen 2002). Novel pathogens via introduced animals can therefore cause devastating impacts on the ecosystems of oceanic islands.

The Bonin Islands are part of the Ogasawara Archipelago, which is located nearly 1000 km south of mainland Tokyo, Japan. The archipelago supports a unique ecosystem involving a wide variety of indigenous species (Momiyama 1930; Shimizu 2003; Kawakami 2008; Kawakami and Okochi 2010) and also serves as an important breeding area for seabirds (Chiba et al. 2007; Kawakami and Okochi 2010). In 2011, the Ogasawara Archipelago was designated as a World Natural Heritage site for its unique ecosystem (UNESCO 2011). Meanwhile, the Bonin grosbeak ( *Carpodacus ferreorostris* ) and Bonin thrush ( *Zoothera terrestris* ), which are both extinct species previously endemic to the Bonin Islands, are thought to have been driven into extinction by introduced rats and cats (BirdLife International 2020). Furthermore, major population declines of endemic species have been recorded through direct and indirect effects of introduced species such as flatworms, lizards, plants, and cats. For these reasons, along with other human‐related activities, many island species have decreased in numbers (Tomiyama 1998; Shimizu 2003; Kawakami 2008; Kawakami and Okochi 2010; Sugiura 2016). Endemic species and subspecies, including the Bonin white‐eye ( *Apalopteron familiare* ) and Bannerman's shearwater ( *Puffinus bannermani* ), have consequently been designated as national endangered species (Ministry of the Environment 2020). Conservation plans have been organized along with conservational research in order to preserve this vulnerable ecosystem (Takahashi 1973; Shimizu 2003; Kawakami and Higuchi 2003; Toma and Miyagi 2005; Takaoka and Saito 2006; Chiba et al. 2007; Yabe et al. 2009; Kawakami and Okochi 2010; Chiba and Suzuki 2011; Sugita, Kawakami, and Nishiumi 2016; Saitoh et al. 2020). However, few studies have addressed the presence and impact of wildlife diseases on the islands, with only *Angiostrongylus cantonensis* in invasive rodents, trematodes in fishes, *Phellinus noxius* in plants, and *Salmonella* (Tokiwa et al. 2013; Sahashi et al. 2015; Kuramochi 2018; Sumiyama et al. 2020). Bloodsucking arthropod insects, including mosquitoes, blackflies, and biting midges, have been confirmed in the Bonin Islands (Takahashi 1973; Tanaka, Mizusawa, and Saugstad 1979; Wada 1986; Takaoka, Saito, and Suzuki 1999; Toma and Miyagi 2005). The presence of both endemic and introduced avian species, along with possible vector species, creates a similar scenario to other oceanic islands in which avian malaria parasites and APV have had significant impacts. As part of conservational research, we investigated the prevalence of avian haemosporidia and APV in the Bonin Islands.

## 2 Materials and Methods

### 2.1 Study Site

**FIGURE 1:**
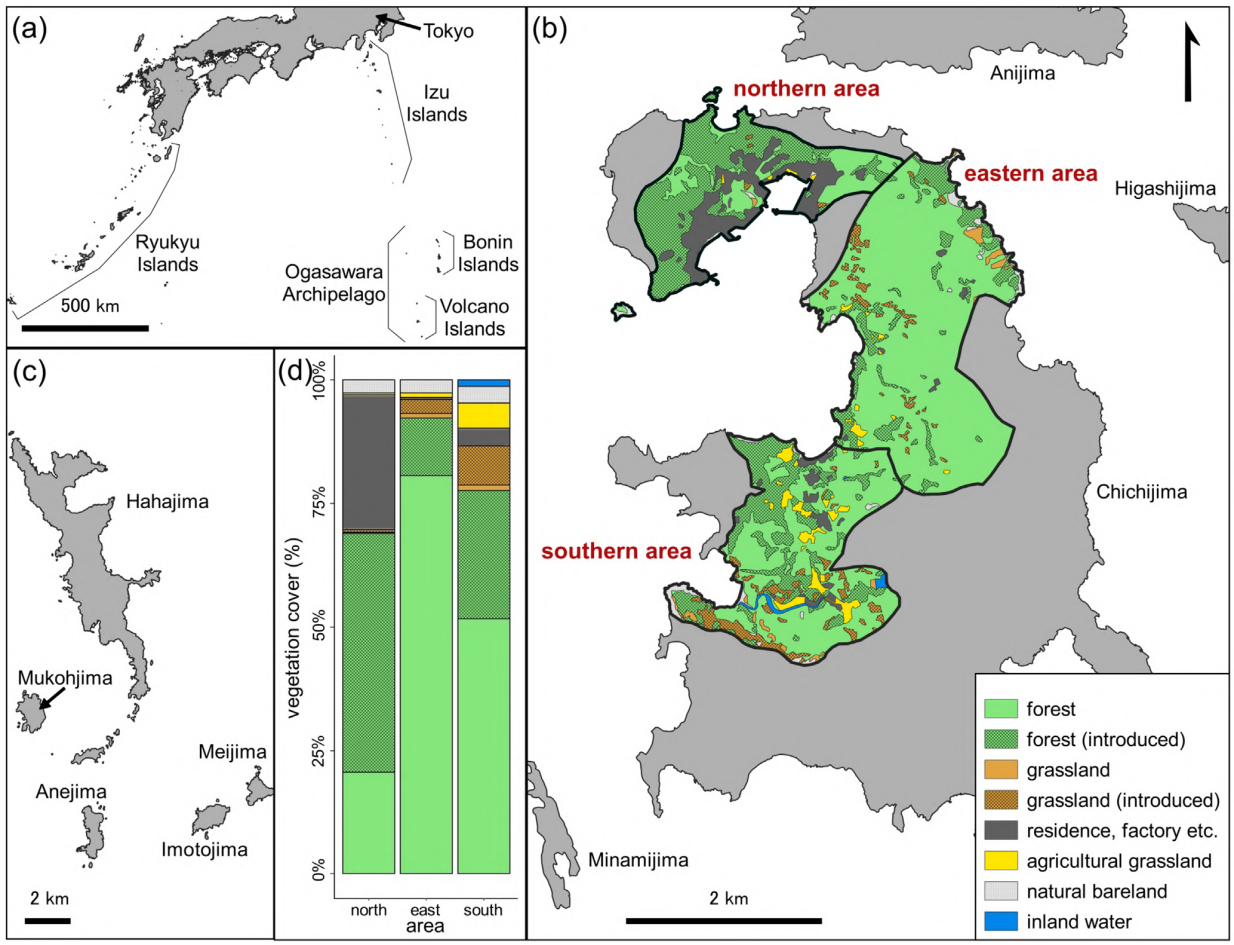
Map of the study area. Location of the Bonin Islands in relation to other areas of Japan (a). Islands of the Chichijima group, with vegetation cover of the three sampling areas (b). Islands of the Hahajima group (c). Vegetation cover proportions of the three sampling areas (d).

Birds and mosquitoes were sampled in the Bonin Islands (Figure 1a). Sampling took place in islands of the Chichijima group (Figure 1b): Chichijima (27°04′N, 142°13′E), Anijima (27°07′N, 142°12′E), Minamijima (27°02′N, 142°10′E), and Higashijima (27°06′N, 142°15′E) and islands of the Hahajima group (Figure 1c): Hahajima (26°40′N, 142°09′E), Mukohjima (26°36′N, 142°07′E), Meijima (26°34′N, 142°13′E), Imotojima (26°33′N, 142°12′E), and Anejima (26°33′N, 142°09′E). These islands are located in the subtropical climate zone. The mean annual temperature of Chichijima is 23°C with a narrow range of 18°C–28°C. The annual precipitation is about 1300 mm, with the most rainfall in May (174 mm) and the least in February (61 mm) (Ministry of the Environment 2010; Japan Meteorological Agency 2020). The archipelago is low‐lying, with the highest elevations on Chichijima and Hahajima being 326 and 462 m, respectively (Government of Japan 2010). Currently, most of the areas are strictly protected, and only parts of Chichijima and Hahajima are inhabited by people (Ministry of the Environment 2010).

Samples of Chichijima were collected in three main areas, classified according to the city they were sampled (Figure 1b). Vegetation cover proportions differ among these areas (Figure 1d), as calculated from vegetation maps using Quantum GIS ver. 3.14 (Biodiversity Center of Japan 2016; National Statistics Center 2018; QGIS Developmental Team 2020). The northern area is the main residential area of Chichijima, surrounded by secondary forests consisting of mainly introduced species such as *Casuarina equisetifolia* and *Leucaena leucocephala* communities. The southern area is a smaller residential area, surrounded by agricultural grasslands as well as introduced and natural forests. Human settlement and introduced species have had great impacts on the northern and southern areas. In contrast, the vegetation in the eastern area of sampling is mostly natural and well preserved. The area is mostly covered by *Machilus kobu*‐*Schimetum mertensianae* communities, which are currently severely managed for conservation measures. Information on the precise location of collection for many birds could not be obtained, and hence details on the habitat during collection were unknown.

### 2.2 Sampling Rescued or Deceased Birds

Birds were collected in the Bonin Islands between 2011 and 2019. These include birds that were found dead and rescued birds that died during rehabilitation. 378, two, one, seven, and five bird(s) were found in Chichijima, Anijima, Higashijima, Minamishima, and Hahajima, respectively. The sampling size was particularly large in Chichijima, where the NPO Institute of Boninology actively collected deceased birds. Note that resident bird species were not collected in Higashijima and Minamishima. Additionally, one bird landed on the passenger ship Ogasawara‐maru, which travels between Tokyo and Chichijima. For six birds, a portion of the liver and heart were sampled prior to freezing, and impression smears were prepared from the cut surface of the heart. All other individuals were frozen prior to necropsy. Tissue samples were collected from the liver, lung, heart, or muscle and placed in microtubes containing 70% ethanol directly after necropsy. In eight warbling white‐eyes and one White's thrush ( *Zoothera aurea* ), APV‐like lesions were seen on the eyelids, nares, legs, and wing joints. These lesions were removed, placed in microtubes, and kept at −20°C until further processes. For one warbling white‐eye, a lesion from the leg was placed in a microtube containing 10% neutral buffered formalin.

In addition, blood from three birds rescued in Chichijima was sampled at the Institute of Boninology in August of 2015. Blood was drawn from the brachial or jugular vein, and blood smears were prepared. The remaining blood was placed in microtubes containing 70% ethanol.

Impression smears and blood smears were fixed with methanol and stained with Hemacolor (Merck KGaA, Darmstadt, Germany). Once dry, the smears were mounted in Eukitt medium (O. Kindler GmbH, Freiburg, Germany). The formalin‐fixed lesion was embedded in paraffin to make thin sections, which were mounted on slides and stained with hematoxylin and eosin.

### 2.3 Sampling Birds by Mist‐Netting

Resident passerines were captured with mist nets in February 2016, September 2020, and June to July 2021. 38 and 89 birds were captured in Chichijima and the Hahajima group [Hahajima (*n* = 3), Mukohjima (*n* = 36), Meijima (*n* = 11), Imotojima (*n* = 4), and Anejima (*n* = 35)]. Blood was obtained from the brachial vein and placed in microtubes containing 70% or 99% ethanol. Blood smears were also prepared for individuals captured in 2016 and 2021. The smears were prepared and stained in the same manner as rescued birds. The birds were released after they were ringed.

### 2.4 DNA Extraction and Molecular Detection of Avian Haemosporidia From Birds

DNA was extracted from organ tissue and blood samples using the standard phenol‐chloroform method, with tris‐EDTA as the final buffer. DNA concentration was confirmed with Nanodrop One Microvolume UV–Vis Spectrophotometers (Thermo Fisher Scientific, MA, USA) and adjusted to a final concentration of 50 ± 10 ng/μL. A nested PCR targeting the partial mitochondrial cytochrome *b* (cyt*b*) gene of avian malarial parasites (*Plasmodium*) and related parasites (*Haemoproteus* and *Leucocytozoon*) was performed using the primers HaemNFI/HaemNR3 for the first PCR and HaemF/HaemR2 and HaemFL/HaemR2L for the second PCR (Hellgren, Waldenström, and Bensch 2004). The reaction mixture composition was as follows: 2 mM MgCl_2_, 0.2 mM deoxynucleotide triphosphate, 10x ExTaq buffer (Mg^2+^ free; Takara, Ohtsu, Japan), 0.625 U Ex‐Taq (Takara), 0.6 μM each primer, and 50 ng of template DNA, making the final volume 25 μL each. *Plasmodium gallinaceum* GALLUS01 from an experimentally infected chicken ( *Gallus gallus* ) and *Leucocytozoon* sp. OTULEM04 from a rescued Japanese scops‐owl ( *Otus semitorques* ) were included as positive controls. For a negative control, one sample containing distilled water instead of DNA was also included. Amplification was confirmed on 1.5% agarose gels (Agarose S; Nippon Gene, Tokyo, Japan) containing ethidium bromide (Nacalai Tesque, Kyoto, Japan), which were placed in chambers filled with TAE buffer. One positive control and one negative control were included in every gel. Electrophoresis was performed at 100 V for approximately 20 min. Gels were placed under ultraviolet light to confirm amplification. If any bands were seen in the negative control, all samples in the same gel were retested from the first PCR. Positive bands were cut out of the gel, and DNA was extracted using thermostable β‐agarase (Nippon Gene, Tokyo, Japan).

Additionally, for all samples positive for *Plasmodium* or *Haemoproteus* using the above primers, a nested multiplex PCR was carried out in order to detect possible co‐infections (Pacheco et al. 2018). First, PCR targeting a portion of the mitochondrial *COX1* gene and the full cyt*b* gene was performed with the primers AE298/AE299. For the second PCR, the primers AE983/AE985 and AE980/AE982 were used to detect *Plasmodium* and *Haemoproteus*, respectively. Positive controls consisted of *P. gallinaceum* GALLUS01 from an experimentally infected chicken and *H. larae* SPMAG12 from a captive African penguin ( *Spheniscus demersus* ). Positive bands for *Plasmodium* were cut out, and DNA was extracted using thermostable β‐agarase (Nippon Gene, Tokyo, Japan). If positive bands for *Haemoproteus* were detected, an additional PCR was carried out with the primers HaemF/AE982 (Hellgren, Waldenström, and Bensch 2004; Pacheco et al. 2018), and positive bands were cut out and extracted. Reaction mixture compositions and all electrophoresis processes were the same as above. PCR conditions were according to protocol (Pacheco et al. 2018).

### 2.5 Sampling Mosquitoes

Mosquitoes were collected in Chichijima by sweep netting (March and August 2015) or CDC traps enhanced with yeast‐generated CO_2_ (July and August 2015) (Tsuda et al. 2009). Sweep netting was carried out in the morning or evening at five, two, and three localities in the northern, eastern, and southern areas, respectively. Two traps each were set in the northern and southern areas. Traps were set in the evening and recovered in the morning. Captured mosquitoes were morphologically identified to a species (Tanaka, Mizusawa, and Saugstad 1979; Tsuda 2019) and then placed in −20°C until further processes.

### 2.6 DNA Extraction and Molecular Detection of Avian Haemosporidia From Mosquitoes

Mosquitoes were separated into head‐thorax and abdomen under an Olympus SZ61TR stereo microscope (Olympus, Tokyo, Japan). DNA was extracted from each sample individually, using the REDExtract‐N‐Amp Tissue PCR kit (SIGMA, St. Louis, MO, USA). To detect *Plasmodium*/*Haemoproteus* parasites, nested PCR targeting the partial cyt*b* gene of avian haemosporidia was performed. We used different primer sets from birds as a result of some trials to more effectively amplify the parasite DNA from mosquitoes. The primers DW2/DW4 were used for the first PCR (Perkins and Schall 2002), followed by a second PCR using HaemNF/HaemNR2 (Waldenström et al. 2004). The composition of the reaction mixture was as follows: 4 mM MgCl_2_, 0.4 mM deoxynucleotide triphosphate, 10x ExTaq buffer (Mg^2+^ free; Takara, Ohtsu, Japan), 1 U Ex‐Taq (Takara), 0.4 μM each primer, and 1 μL of template DNA, making the final volume 25 μL each. Cycle conditions were as previously described (Perkins and Schall 2002; Waldenström et al. 2004). *Plasmodium gallinaceum* GALLUS01 and distilled water were used for the positive and negative controls, respectively. Electrophoresis was carried out in the same method as the avian samples.

### 2.7 Phylogenetic Analysis of Avian Haemosporidian Lineages

The extracted DNA was sequenced in both directions with the BigDye terminator cycle sequence kit 3.1 (Applied Biosystems, Foster City, CA, USA) and the ABI 3130‐Avant Auto Sequencer (Applied Biosystems). If double‐base callings were detected, the sample was retested on a different day using newly extracted DNA. A sample was considered co‐infected if double‐base callings were seen in both detections. Obtained sequences were assembled and compared with sequences in the GenBank database (Madden 2013) and MalAvi database (Bensch, Hellgren, and Pérez‐Tris 2009). If lineages identical to the positive controls were detected, samples were retested from the first PCR to remove contaminations.

Mr. Bayes version 3.2 was used to construct a Bayesian phylogeny (Ronquist and Huelsenbeck 2003). Morphologically identified lineages of the three haemosporidian genera and molecularly close lineages were included for comparison. *Theileria annulata* was selected as an outgroup. The Jukes‐Cantor model of substitution was implemented to calculate genetic distances between lineages using MEGA X (Kumar et al. 2018). Using the same software, translated amino acids were also compared between lineages. Using ModelFinder in IQ‐TREE 1.6.12 (Kalyaanamoorthy et al. 2017), the General Time Reversible model with gamma distribution for variable sites and proportion of sites as invariable (GTR+Γ+I) was selected as the best‐fit model under the Bayesian information criterion. Markov chain Monte Carlo (MCMC) sampling was run twice independently for three million generations, with a sampling frequency of every 1000 generations (Ronquist et al. 2012). The first 25% of trees were discarded as “burn‐in,” and the resulting tree was visualized with FigTree 1.4 (Rambaut 2012). A minimum spanning network was generated with PopART 1.7 (Leigh and Bryant 2015) to visualize genetic relationships between the obtained lineages.

### 2.8 DNA Extraction and Molecular Detection of APV

DNA was extracted from APV‐like lesions using the DNeasy blood & tissue kit (Qiagen, GmbH, Hilden, Germany). A partial sequence of the 4b core protein gene was amplified by PCR using the primers P1x and P2 (Huong et al. 2014). The reaction mixture composition was as the following: 2.5 mM MgCl_2_, 0.4 mM deoxynucleotide triphosphate, 10× ExTaq buffer (Mg^2+^ free; Takara, Ohtsu, Japan), 1 U Ex‐Taq (Takara), 0.4 μM each primer, and 50 ng of template DNA, making the final volume 25 μL each. Cycle conditions were according to protocol (Huong et al. 2014). Fowl pox vaccine (Nisseiken Co. Ltd., Tokyo, Japan) was used as a positive control. The negative control contained distilled water instead of DNA. Procedures from electrophoresis to sequence assembly were carried out in the same methods as avian haemosporidian detection. Obtained sequences were compared with sequences in the GenBank database using BLAST (Madden 2013). Genetic distances were calculated by implementing the Jukes‐Cantor model of substitution in MEGA X (Kumar et al. 2018).

### 2.9 Phylogenetic Analysis of APV Strains

A phylogeny was generated using Mr. Bayes version 3.2 (Ronquist et al. 2012), including sequences from previous studies (Gyuranecz et al. 2013; Banyai et al. 2015; Sarker et al. 2017) and unpublished sequences in the Nihon University College of Bioresource Sciences Laboratory of Biomedical Science database. The transition model with gamma distribution for variable sites and proportion of sites as invariable (TIM+Γ+I) was selected as the best‐fit model under the Bayesian information criterion using ModelFinder implemented in IQ‐TREE 1.6.12 (Kalyaanamoorthy et al. 2017). As TIM is not implemented in Mr. Bayes version 3.2, the substitution model was replaced with General Time Reversible (GTR) model. Markov chain Monte Carlo (MCMC) sampling was run twice independently for three million generations, and samples were taken every 1000 generations. The first 25% of the trees were discarded as a “burn‐in” step. The resulting phylogenetic tree was visualized using FigTree 1.4 (Rambaut 2012).

### 2.10 Microscopic Examinations

Blood smears, impression smears, and tissue sections were observed under an Olympus BX43 light microscope (Olympus, Tokyo, Japan). Blood smears and impression smears were screened at 400× and 1000× magnification. Detected parasites were identified based on their morphological features (Valkiūnas 2005). The tissue section was observed at 200× and 600× magnification. Photographs were taken using an Olympus IX71 light microscope (Olympus) and software cellSens Standard 1.6 (Olympus).

### 2.11 Statistical Analysis

Using Fisher's exact test, the prevalence of avian haemosporidia was compared between resident and migratory species. Migratory status was determined based on the Check‐list of Japanese birds, 7th revised edition (Ornithological Society of Japan 2012), as well as field studies on the avifauna of the Bonin Islands (Chiba et al. 2007; Emura 2011; Kawakami et al. 2018). To investigate variables that affect *Plasmodium* prevalence within the islands, general linear models (GLM) with binomial distribution and logit function were used. Species (warbling white‐eye, blue rock thrush, or White's thrush), age (juvenile or adult), sampling area (north, east, or south), and season of sampling (spring, summer, autumn, or winter) were tested. Only samples from Chichijima were included in the GLM tests. Other resident species were not included due to the small sample size. Furthermore, to avoid potential bias due to parasite lineage variation, only individuals positive for GRW06 were included. For ANOVA calculations, the type II sums of squares were used because of unbalanced data. Tukey's test for multiple comparisons was used for post hoc comparisons. For *Cx. boninensis* mosquitoes, the effect of sampling area on GRW06 prevalence was tested with Fisher's exact test. All statistical analyses were done using the software R version 3.6.3 (R Core Team 2020), using packages “prevalence,” “car,” and “multcomp” (Hothorn, Bretz, and Westfall 2008; Fox and Weisberg 2011; Devleesschauwer et al. 2014). Statistical significance was determined using the 5% significance level.

## 3 Results

### 3.1 Avian Haemosporidia in Birds

Avian haemosporidian DNA was detected from 209 of the 524 examined birds (Table 1). Among resident species, positive individuals were detected from Chichijima, Hahajima, Mukohjima, Meijima, Imotojima, and Anejima (Supporting Information S1). In migratory species, five individuals from Chichijima were positive by PCR. No positive individuals were detected from the remaining three islands (Anijima, Higashijima, Minamijima). The overall prevalence was significantly higher in resident species (53.68%; 95% CI = 48.66%–58.64%) compared to migratory species (3.47%; 95% CI = 1.49%–7.87%) (Fisher's exact test: *p* < 0.001).

**TABLE 1:** Summary of birds sampled in this study, with PCR results of haemosporidian detection.

Status		Chichijima group		Hahajima group		Total		Lineages^a^
		Sampled	Infected^b^	Sampled	Infected^b^	Sampled	Infected^b^	
Resident		290	149 (51.38)	90	55 (61.11)	380	204 (53.68)	pGRW04, pGRW06, pCXPIP12, pMONTRI01, hZOSJAP02^c^, hZOOLUN01, lZOOAUR01^c^, lZOOAUR02^c^, lZOOAUR03^c^, *Leucocytozoon* sp. [co‐infected]
Migrant	Migrant breeder	88	0	2	0	90	0	
	Winter visitor	34	2 (5.88)	1	0	35	2 (5.71)	pSW2, *Plasmodium* sp. [co‐infected]
	Passage visitor	4	0			4	0	
	Irregular or accidental visitor	14	3 (21.43)	1	0	15	3 (20.00)	pACCBAD01, pGRW06, lANACRE04^c^
	Sub‐total	140	5 (3.57)	4	0	144	5 (3.47)	
	Total	430	154 (35.81)	94	55 (58.51)	524	209 (39.89)	

^a^ Lineage names are given according to MalAvi database.

^b^ Parentheses: prevalence.

^c^ Lineages detected for the first time.

**FIGURE 2:**
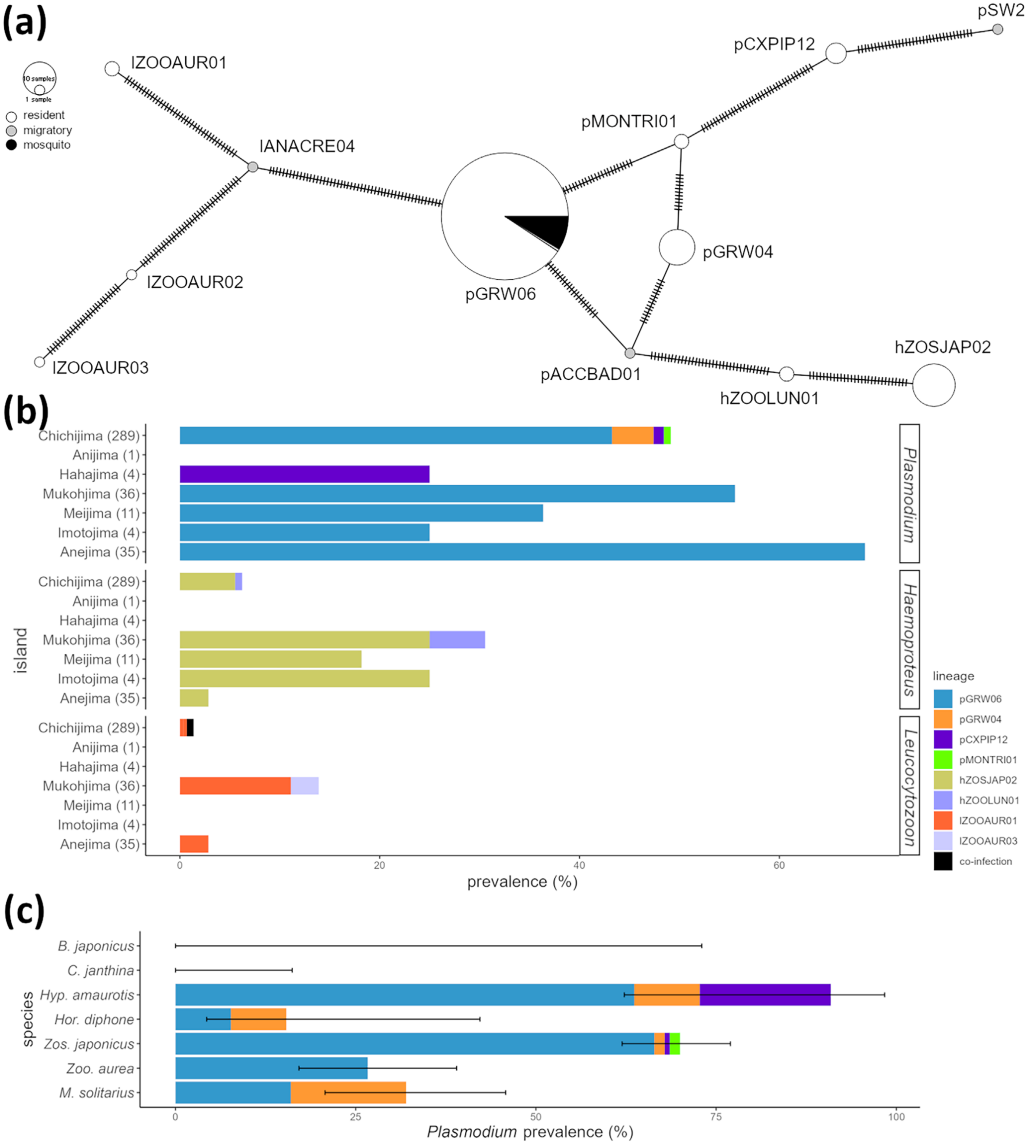
Composition of haemosporidian mitochondrial DNA cytochrome *b* lineages detected in this study. Minimum spanning network (a), prevalence among resident species by island (b), and *Plasmodium* prevalence among resident species of Chichijima (c). The colors represent host/vector status (a) or lineages (b, c), as shown in the legends. Islands or species lacking bars show 0% prevalence (i.e., no parasites were detected; b, c). Each hash mark indicates a base mutation (a). Error bars represent ±95% of the confidence interval (c). Note that two species have 0% prevalence but show a long error bar due to the small sample sizes (c).

A total of six *Plasmodium*, two *Haemoproteus*, and four *Leucocytozoon* lineages were detected in this study, including five lineages detected for the first time (Table 1, Figure 2, Supporting Information S1). All detected lineages varied from one another by more than 10 bases within the observed genetic region (Figure 2a). Detected *Haemoproteus* lineages were placed in the clade of parasites belonging to the subgenus *Parahaemoproteus* (Supporting Information S1). Three birds were co‐infected with parasites of the same genus, and lineages could not be determined. In 27 warbling white‐eyes, the primers HaemF/HaemR2 only detected *Plasmodium* parasites, but both *Plasmodium* and *Haemoproteus* parasites were detected using the genus‐specific primers (Supporting Information S1). For *Plasmodium*, HaemF/HaemR2 and the genus‐specific primers detected the same lineages (26 pGRW06 and 1 pMONTRI01). The single lineage hZOSJAP02 was detected from all 27 individuals using the *Haemoproteus*‐specific primers. The only lineage shared between resident and migratory species was pGRW06 of *Plasmodium elongatum*.

Nine lineages were detected from resident birds in Chichijima (*n* = 8), Hahajima (*n* = 1), Mukojima (*n* = 5), Meijima (*n* = 2), Imotojima (*n* = 2), and Anejima (*n* = 3). (Figure 2b, Supporting Information S1). Five lineages (pGRW06, pCXPIP12, hZOSJAP02, hZOOLUN01, and lZOOAUR01) were detected from multiple islands, all of which were detected in both the Chichijima and Hahajima groups.

Parasites were detected from six of the eight resident bird species (Table 1). *Plasmodium elongatum* (pGRW06) was the most prevalent parasite species among all three parasite genera and within *Plasmodium* lineages (Figure 2b). The second most prevalent *Plasmodium* species was *P. relictum* (pGRW04), although detected far less frequently. These two parasite species were detected across multiple resident bird species of Chichijima, although the prevalence greatly differed between host species (Figure 2c, Supporting Information S1). 27 and 10 resident birds were positive for *Haemoproteus* spp. and *Leucocytozoon* spp., respectively.

Among the tested individuals, age was not a significant factor regarding pGRW06 prevalence in Chichijima (GLM: LR *χ* ^2^ = 1.372, df = 1, *p* = 0.242), and no interactions were detected. Meanwhile, bird species (GLM: LR *χ* ^2^ = 35.153, df = 2, *p* < 0.001) was a significant factor, with a significant interaction with season (GLM: LR *χ* ^2^ = 18.431, df = 6, *p* = 0.005). No seasonal variations in prevalence were seen in White's thrush (GLM: LR *χ* ^2^ = 5.003, df = 3, *p* = 0.172; GLM (northern area only): LR *χ* ^2^ = 3.455, df = 3, *p* = 0.327) and blue rock thrush (GLM: LR *χ* ^2^ = 0.552, df = 3, *p* = 0.907; GLM (northern area only): LR *χ* ^2^ = 0.833, df = 3, *p* = 0.841), even when compared among only northern samples. Few individuals were sampled in the eastern and southern areas for these two species, and comparing the parasite prevalence among areas was not possible. For the warbling white‐eye, pGRW06 prevalence significantly differed between seasons when all areas were included (GLM: LR *χ* ^2^ = 22.22, df = 3, *p* < 0.001). However, prevalence did not differ among seasons when analyzed separately by area (Table 3). Furthermore, pGRW06 prevalence was higher in the southern area compared to the northern area during the summer and winter, exhibiting a significant difference in prevalence (Tukey's post hoc test for winter: estimate = 2.749, SE = 1.168, *z* value = 2.353, *p* < 0.037). The prevalence was considerably higher in the southern area during the autumn as well, although not significantly (Table 3). The prevalence during the winter was higher in the eastern area compared to the northern area, but the small sample size did not allow for accurate comparisons (Tukey's post hoc test: estimate = −19.096, SE = 3584.671, *z* value = −0.005, *p* = 1.000).

Of the 100 birds in which smears were obtained, parasites were detected by microscopy from 71 birds (Supporting Information S1). Parasites were not found in the smears of six PCR‐positive individuals. Although some parasites could not be identified to a species, *P. elongatum* , *P. relictum*, and *H. killangoi* were present in many smears. Co‐infections of *P. elongatum* and *H. killangoi* were seen in 21 individuals. These microscopy results corresponded to multiplex PCR results, allowing the lineage hZOSJAP02 to be identified as the morphological species *H. killangoi* (Supporting Information S1). However, note that in two individuals, both *P. elongatum* and *H. killangoi* were observed by microscopy, but only *P. elongatum* was detected molecularly. Similarly, co‐infections of *P. elongatum* and *P. relictum* were observed in 2 individuals, but only pGRW06 of *P. elongatum* was detected by both PCR protocols.

### 3.2 Avian Haemosporidia in Mosquitoes

In total, 262 mosquitoes of five species were captured (Table 2). Twelve *Culex boninensis* and one *Cx. quinquefasciatus* caught by sweep netting were PCR‐positive for *P. elongatum* (pGRW06). Specifically, pGRW06 was detected from the head‐thorax and abdomen, head‐thorax, and abdomen of five, one, and six unfed *Cx. boninensis*, respectively, and the total prevalence was 27.27% (95% CI = 16.35%–41.85%). Infected individuals were caught in both March and August. The eastern area had the highest prevalence (31.43%, 95% CI = 18.55%–47.98%), followed by the northern area (20.00%, 95% CI = 3.62%–62.45%). No positive mosquitoes were found in the southern area. The difference between areas was not statistically significant (Fisher's exact test: *p* = 0.592). Prevalence in *Cx. quinquefasciatus* was 1.28% (95% CI = 0.23%–6.91%), and pGRW06 was detected from the abdomen of an unfed female caught in the northern area. In addition, *Haemoproteus* DNA was detected from the abdomen of an unfed *Cx. boninensis*. The parasite lineage could not be determined due to co‐infection.

**TABLE 2:** Mosquito samples collected and tested, including PCR results of haemosporidian detection.

Method	Species	Northern area		Eastern area		Southern area		Total	
		No.	positive	No.	Positive	No.	Positive	No.	Positive
Sweep netting^a^	*Aedes savoryi* ^b^	4/0	0	1/0	0	52/0	0	57/0	0
	*Aedes albopictus*	5/41	0	1/28	0	1/1	0	7/70	0
	*Culex boninensis* ^b^	1/1	1/0	0/35	0/11	3/0	0	4/36	1/11
	*Culex quinquefasciatus*	1/1	0	0/1	0			1/2	0
	*Culex* sp.	1/0	0					1/0	0
	*Lutzia shinonagai* ^b^			0/1	0			0/1	0
	*Lutzia* sp.	1/0	0					1/0	0
	Sub‐total	13/43	1/0	2/65	0/11	56/1	0	71/109	1/11
CO_2_ trap	*Aedes albopictus*	2	0			1	0	3	0
	*Culex boninensis* ^b^	3	0			1	0	4	0
	*Culex quinquefasciatus*	10	1			65	0	75	1
	Sub‐total	15	1			67	0	82	1
	Total	71	2	67	11	124	0	262	13

^a^ March/August.

^b^ Endemic species.

### 3.3 APV In Birds

**FIGURE 3:**
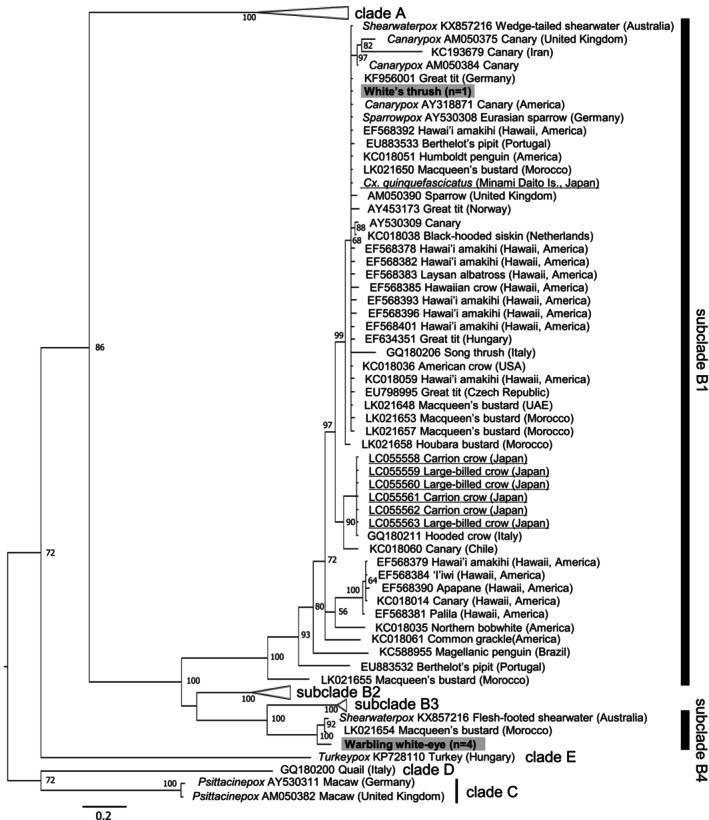
Bayesian phylogenetic analysis of the 4b core protein strains (426 bp) of *Avipoxvirus*. Posterior probabilities of > 0.60 were indicated. Clade A, subclade B2, and subclade B3 were collapsed. Strains previously detected from Japan are underlined. Dark gray background shows strains detected in this study.

All nine tested individuals were PCR‐positive for APV. Features characteristic of APV infection were also seen in morphological observations (Supporting Information S1). Molecular analysis revealed two novel strains, both placed in clade B by phylogenetic analysis (Figure 3). All eight warbling white‐eyes had the same strain of subclade B4, while the White's thrush strain was placed in subclade B1. By pairwise analysis, a strain detected from a Macqueen's bustard ( *Chlamydotis macqueenii* ) (divergence: 1.89% compared within 426 bp) and SWPV1 of *Shearwaterpox* from flesh‐footed shearwaters (*Ardenna carneipes*) (divergence: 2.84% compared within 570 bp) were closest to the warbling white‐eye strain. The White's thrush strain was closest to a strain from a great tit ( *Parus major* ) of Germany (divergence: 0.17% compared within 570 bp), although many other strains in subclade B1 were also very close. Avian malaria parasites were detected from all nine APV‐positive individuals. Specifically, pGRW06 was detected from seven warbling white‐eyes and one White's thrush, and pCXPIP12 was detected from one warbling white‐eye.

## 4 Discussion

### 4.1 Prevalence of Avian Haemosporidia Among Resident Species

Avian haemosporidia was detected from six of the eight resident species tested in this study. Total parasite prevalence and prevalence per lineage widely differed between species. Interestingly, two warbling white‐eyes were co‐infected with *P. elongatum* and *P. relictum* , although only *P. elongatum* GRW06 was detected using two PCR protocols. The difficulty of detecting co‐infections, particularly those of the same genus, has been previously addressed (Bernotiene et al. 2016; Pacheco et al. 2018). Blood smears were obtained for only some individuals, and *P. relictum* prevalence may possibly be higher than the obtained PCR results.

Among resident bird species of Chichijima, there was a significant difference in *P. elongatum* (pGRW06) prevalence (Figure 2c). What appeared to be seasonal variations in the warbling white‐eye was due to variations in sampling area, as separate analysis of northern and southern areas revealed no seasonal variations (Table 3). The pGRW06 prevalence in this species was significantly higher in the southern area compared to the northern area. Additionally, although not statistically significant, all individuals from the eastern area were positive for pGRW06. Meanwhile, positive mosquitoes were caught mostly in the eastern area, although the small sample sizes prevented statistical significance. A large proportion of the northern area is residential land, while the eastern area is covered by mostly natural vegetation (Figure 1b,d). The southern area contains a small residential area, but there is a mixture of different vegetation types. *Cx. boninensis* can be found in various environments but prefers vegetated areas over residential areas, especially areas with vegetation diversity (Maekawa et al. 2021). Warbling white‐eyes in the Bonin Islands utilize a variety of environments, with a preference towards open habitats (Kawakami and Higuchi 2003). Higher parasite prevalence has been recorded in natural habitats (Huang et al. 2015; Ferraguti et al. 2016), in relation to higher vector and host abundance (Bonneaud et al. 2009; Lalubin et al. 2013; Fecchio et al. 2017). The population density of both warbling white‐eyes and *Cx. boninensis* is thought to be high in the eastern and southern areas, increasing contact between the two and therefore increasing transmission. However, other studies revealed no relationships or inverse relationships between host abundance and parasite prevalence (Fecchio et al. 2017; Van Hoesel et al. 2019). Furthermore, sample sizes were small for some areas and seasons, and re‐analysis using a larger data set would be needed to clarify these results. Differences in prevalence among areas could not be tested in other species, and reasons for the variation in parasite prevalence among host species could not be fully revealed.

**TABLE 3:** Prevalence of *Plasmodium elongatum* (pGRW06) in warbling white‐eyes ( *Zosterops japonicus* ) at each sampling area of Chichijima.

Season	North		East		South		LR chisq	*p*
Spring (March to May)	7/21	(33.33)^a^	0		0		—	—
Summer (June to August)	25/43	(58.14)	0		10/11	(90.91)	4.879	**0.027**
Autumn (September to November)	7/15	(46.67)	0		2/2	(100)	2.781	0.095
Winter (December to February)	8/13	(61.54)	9/9	(100)	25/26	(96.15)	10.370	**0.006**
LR chisq	4.245				0.598			
*p*	0.236				0.742			

*Note:* Significant *p* values are shown in bold.

^a^ Positive/sampled (prevalence by percent).

### 4.2 Transmission of Avian Haemosporidia Within the Bonin Islands

A total of nine haemosporidian lineages were detected from resident birds and are therefore thought to be transmitted within the archipelago. The most prevalent lineage, pGRW06, was also detected from *Culex boninensis* and *Cx. quinquefasciatus* of Chichijima. In *Cx. boninensis*, pGRW06 was detected from both the head‐thorax and abdomen of unfed individuals. Considering the life cycle of *Plasmodium* parasites, detection from the head‐thorax and abdomen would indicate the presence of sporozoites in the salivary gland and oocysts in the mid‐gut, respectively (Valkiūnas 2005). This suggests that *Cx. boninensis* may be a competent vector species of pGRW06. However, some *Plasmodium* species undergo abortive sporogonic development in incompetent vectors (Žiegytė 2014; Žiegytė and Valkiūnas 2014), and further studies would be required to prove vector competency. *Cx. quinquefasciatus* is a competent vector of *P. elongatum* (pERIRUB01) and may also be competent for pGRW06. However, parasite intensity, environmental conditions, and parasite lineage may affect the competency (Palinauskas et al. 2016). *Cx. quinquefasciatus* has been experimentally confirmed to be a competent vector of *P. relictum* (pGRW04) (Lapointe, Goff, and Atkinson 2005; Valkiūnas et al. 2015). *Plasmodium* sp. CXPIP12 has been molecularly detected from unfed *Cx. pipiens* (Kim and Tsuda 2010), a closely related species of *Cx. quinquefasciatus*. While further studies will be necessary to confirm vector competency, our results suggest that *Cx. boninensis* and *Cx. quinquefasciatus* are likely to be important vector species of avian malaria in Chichijima. Positive mosquitoes were caught in both March and August, suggesting that if competent, transmission is possible as early as March. The prevalence in mosquitoes (27.27%) was high compared to previous studies ranging from 0.04% to 15.4% (Ferraguti et al. 2024), although reasons are unknown and hope to be investigated in future studies.

Infected resident birds were detected in both the Chichijima group and Hahajima group, suggesting that transmission is possible in both areas. *Cx. boninensis* and *Cx. quinquefasciatus* have both been confirmed in Hahajima (Maekawa et al. 2021), suggesting that transmission is possible in Hahajima as well. While five parasite lineages were detected in both island groups, two *Plasmodium* lineages (pGRW04 and pMONTRI01) were detected from only Chichijima, and lZOOAUR03 was detected from only Mukohjima. Host species of these lineages were investigated in both island groups, and parasite fauna may differ due to limited movement of individuals among the island groups. However, the number of investigated individuals widely differs between islands and island groups. Further sampling may result in a more complete representation of the parasite fauna of each island.

*Haemoproteus* (subgenus *Parahaemoproteus*) parasites and *Leucocytozoon* parasites are generally known to be transmitted by biting midges (Ceratopogonidae) and blackflies (Simuliidae), respectively (Valkiūnas 2005). The two *Haemoproteus* lineages detected in this study were both placed in the subgenus *Parahaemoproteus* clade (Figure A1), suggesting that they are likely transmitted by biting midges. Both dipteran groups have been confirmed on Chichijima and Hahajima (Wada 1986; Takaoka, Saito, and Suzuki 1999) and may serve as vectors of these two haemosporidian genera. Future investigations of these dipteran groups would be important to better understand the avian haemosporidian transmission within the Bonin Islands. Abortive *Haemoproteus* parasites were detected by PCR in *Culex* mosquitoes for up to 17 days post infection in experimental conditions (Valkiūnas et al. 2013; Žiegytė 2014). In this study, a mixed infection of *Haemoproteus* parasites was detected from one *Cx. boninensis*, in which abortive parasites may have been detected by PCR.

### 4.3 Speculating the Origin of Haemosporidian Parasites in the Bonin Islands

Two and four lineages were detected in only the warbling white‐eye and White's thrush, respectively. Warbling white‐eyes in the Bonin Islands are considered hybrids between *Z. j. alani* of the Volcanic Islands and *Z. j. stejnegeri* of the Izu Islands (Momiyama 1930), although a more recent study suggests populations of the Ryukyu Islands may also be involved (Sugita, Kawakami, and Nishiumi 2016). These subspecies were introduced to the Bonin Islands in the early 1900s (Momiyama 1930), and the resultant hybrids are currently one of the dominant species of the Bonin Islands (Kawakami and Okochi 2010; Sugita, Kawakami, and Nishiumi 2016). Meanwhile, White's thrushes naturally established breeding populations in the mid‐1900s during US occupation, due to the introduction of earthworms (Kawakami 2008, 2019). The lineages solely detected from these species were presumably introduced to the islands along with their host species. While molecular investigations of avian haemosporidia have been made in these surrounding areas (Beadell et al. 2004; Murata 2007; Cannell et al. 2013; Zhang et al. 2014; Huang et al. 2015; Inumaru, Murata, and Sato 2017; Tsuda 2017; Eastwood et al. 2019), the Izu Islands and Volcanic Islands have not been investigated thus far. There are only some studies of avian haemosporidia in the Ryukyu Islands (Hagihara et al. 2005; Nagata 2006; Murata 2007; Murata et al. 2008), few of which have molecular information available (Ejiri et al. 2008; Tanaka et al. 2019). Investigations and molecular comparisons of parasites in surrounding island groups may reveal further insights on the parasite fauna of the Bonin Islands.

*Plasmodium* sp. CXPIP12 was detected from both the warbling white‐eye and brown‐eared bulbul. Like the above‐mentioned lineages, this lineage may have been introduced with warbling white‐eyes. Meanwhile, this lineage was previously detected from brown‐eared bulbuls of mainland Japan and Tsushima, as well as *Cx. pipiens* of mainland Japan (Kim and Tsuda 2010; Tanigawa et al. 2013; Inumaru, Murata, and Sato 2017). The brown‐eared bulbul population of the Bonin Islands is thought to have originated from populations of the Ryukyu Islands (Sugita, Kawakami, and Nishiumi 2016), and the lineage pCXPIP12 may have been naturally introduced with the host.

The most prevalent lineages, pGRW06 and pGRW04, are two of the most widespread avian malaria lineages (Bensch, Hellgren, and Pérez‐Tris 2009; Ellis et al. 2018). Lineages with wide host ranges and geographical distributions tend to be more successful in new areas. In New Zealand, these two lineages were detected from both introduced and native species but were thought to be introduced along with the introduced host species because they were not detected from the neighboring country Australia while frequently detected in the origin of the introduced host species (Ewen et al. 2012). The fact that introduced species came from a distant area makes such analysis possible. Meanwhile, the introduced warbling white‐eyes originate from neighboring island groups, which is the possible origin of other endemic subspecies of the Bonin Islands (Emura et al. 2013; Sugita, Kawakami, and Nishiumi 2016). The overlap makes speculations on the origins of these widespread lineages difficult, and genetic analyses of other genes may be required (Garcia‐Longoria et al. 2015; Hellgren et al. 2015; Huang et al. 2019). Also, endemic mosquitoes, blackflies, and biting midges have been confirmed on the Bonin Islands (Takahashi 1973; Tanaka, Mizusawa, and Saugstad 1979; Wada 1986; Takaoka, Saito, and Suzuki 1999; Toma and Miyagi 2005), including *Cx. boninensis*, from which pGRW06 was detected. The availability of possible vector species demonstrates that avian haemosporidia may have been present much earlier than the introduction of alien avian species, and calibrating the divergence of these arthropod vectors would help to understand when transmission became possible within the islands.

### 4.4 Possibilities on the Introduction of Avian Haemosporidia via Migratory Birds

Collectively, resident birds had a significantly higher parasite prevalence compared to migratory birds (Table 1). A similar case was recently reported, addressing several possible explanations, including parasite host specificity, parasite survival, and host survival (Soares, Latta, and Ricklefs 2020). In our study, one of the primary factors is thought to be the presence of seabirds and waders, which are generally known to have low haemosporidian prevalence (Mendes et al. 2005; Quillfeldt et al. 2010, 2011; Pardal et al. 2014; Clark, Clegg, and Klaassen 2016; Martínez‐De La Puente et al. 2017). Saline environments are not suitable for most vector species, and birds that use these environments are therefore able to minimize exposure to these vectors (Clark, Clegg, and Klaassen 2016). Larvae of some mosquito species, including *Aedes savoryi*, can tolerate saltwater environments (Dale and Knight 2008; Katano et al. 2010; Tsuda 2019). However, *Culex boninensis* breeds in freshwater environments (Toma and Miyagi 2005; Dale and Knight 2008; Tsuda 2019; Maekawa et al. 2021) and may therefore have reduced contact with seabirds. Additionally, taxonomic factors related to immunocompetence and host–parasite assemblages have also been proposed to explain the low parasite prevalence in seabirds (Martínez‐Abraín, Esparza, and Oro 2004). Largely due to the location of the islands, most migratory birds in this study were either seabirds or waders, which partially explains the low prevalence of migratory birds.

In addition to the low prevalence in migratory species, only one lineage was shared between migratory and resident species (Figure 2a). Furthermore, all detected lineages were genetically distant from one another. If lineages were regularly traded among resident and migratory species, shared lineages would be expected between the two. The only shared lineage, pGRW06 of *P. elongatum* , is one of the most widespread *Plasmodium* lineages (Bensch, Hellgren, and Pérez‐Tris 2009) and has been detected in mainland Japan as well (unpublished data). Meanwhile, the lineage is also the most prevalent lineage on Chichijima. It is therefore unknown whether the migrant was infected in the Bonin Islands or elsewhere. The low sharing of lineages may be a result of host specificity, the lack of proper vector species, or habitual segregations between resident and migratory birds (Beadell et al. 2004; Valkiūnas 2005; Hellgren, Pérez‐Tris, and Bensch 2009; Clark and Clegg 2017; Chahad‐Ehlers et al. 2018; Fecchio et al. 2019). Nonetheless, transmission between migratory and resident species, and therefore establishments of new haemosporidian parasites, is thought to be relatively infrequent in the Bonin Islands. However, if all the necessary conditions are satisfied (i.e., an appropriate vector with access to both an infected migratory bird and an uninfected resident bird), introductions of new parasites may be possible, and careful monitoring will be needed in the future.

### 4.5 APV In the Bonin Islands

APV was detected from all nine tested individuals. In the tissue section from a warbling white‐eye, characteristic observations confirmed APV infection (Supporting Information S1). Two different strains of APV were detected in this study (Figure 3). Both strains were placed in the *canarypox*‐like clade B (Gyuranecz et al. 2013). Subclade B1 has the highest host and geographic diversity among all other subclades (Gyuranecz et al. 2013; Le Loc'h et al. 2014; Sarker et al. 2017). Meanwhile, only a few strains of subclade B4 have been detected thus far, but from taxonomically distant species (Le Loc'h et al. 2014; Sarker et al. 2017). APV has generally been considered to be host‐specific to a degree, but is also affected by ecological niche, habitat, and geographical backgrounds (Jarmin et al. 2006; Gyuranecz et al. 2013; Le Loc'h et al. 2014). The two host species in which poxviruses were detected are relatively new species to the Bonin Islands (Kawakami 2008; Sugita, Kawakami, and Nishiumi 2016), as discussed above. However, the relatively relaxed host specificity of APV calls for close monitoring of APV to detect infections of other resident species, as well as shearwaters. The presence of the *canarypox*‐like clade is particularly alarming in relation to the Bonin greenfinch (*Chloris kittlitzi*), an endemic species of the Ogasawara Archipelago. This species is a member of the Fringillidae family, which includes canaries (Gill, Donsker, and Rasmussen 2021). *Canarypox* is highly pathogenic for domestic canaries (Giddens et al. 1971; Shivaprasad et al. 2009), and *canarypox*‐like strains have been detected from other birds of the Fringillidae family (Warner 1968; Pérez‐Tris et al. 2011; Gyuranecz et al. 2013). The *canarypox*‐like strains detected in this study may pose an additional threat to this critically endangered species.

APV can be transmitted by mainly two methods: direct contact of broken skin or indirect contact through mechanical vectors (Aruch et al. 2007; Huong et al. 2014; Lee et al. 2017; Yeo et al. 2019), although other methods of transmission have also been recorded (Kleindorfer and Dudaniec 2006). Warbling white‐eyes, along with other *Zosterops* species, are known to do mutual preening or allopreening (Harrison 1965; Kikkawa and Kakizawa 1981). Such direct contacts between individuals could possibly facilitate the transmission of APV from one individual to another. Alternatively, virions can be transmitted by mechanical vectors, including mosquitoes, biting midges, and mites (Aruch et al. 2007; Huong et al. 2014; Lee et al. 2017; Yeo et al. 2019). All APV‐positive individuals were also positive for avian malaria. In Hawaii, introduced *Cx. quinquefasciatus* are thought to be the main vector of both avian malaria and APV (Warner 1968; Van Riper et al. 1986; Van Riper, Van Riper, and Hansen 2002; Atkinson and Lapointe 2009; Samuel et al. 2018). Similarly, results from this study suggest that APV is transmitted along with avian malaria through mosquitoes, rather than by direct contact. On the other hand, immune functions related to chronic malaria are also thought to play an important role by making birds more susceptible to pox infections (Samuel et al. 2018). This suggests that individuals infected with avian malaria are more likely to develop severe pox infections. Further monitoring may help in understanding the transmission of APV within the Bonin Islands.

## 5 Conclusion

In this study, the presence of avian pathogens of avian malaria and fowl pox was confirmed in the Bonin Islands of Japan for the first time. The existence of endemic resident, introduced, and migratory avian species, as well as endemic mosquitoes, depicts a close scenario to that of New Zealand. Detection of both pathogens from the critically endangered Bonin greenfinch was particularly alarming. Meanwhile, it was not possible to fully understand when and where these pathogens came from, and most islands require additional investigations. In the scope of conservation, it would be crucial to investigate the distribution and transmission status of avian haemosporidia at the island level in order to clarify whether conservation actions would involve specific islands, island groups, or the whole archipelago. We were also unable to investigate the endangered and endemic Bonin white‐eye. Furthermore, pathogenicity and possible impacts on the ecosystem of the Bonin Islands are still unknown. Continuous monitoring, including surrounding islands, will be needed to better understand the impact of these pathogens on the ecosystem of the Bonin Islands.

## Author Contributions

**Mizue Inumaru:** data curation (lead), formal analysis (lead), investigation (lead), methodology (lead), resources (lead), validation (lead), visualization (lead), writing – original draft (lead). **Rui Kimura:** investigation (equal), methodology (equal), validation (equal). **Naoko Suzuki:** resources (supporting). **Hajime Suzuki:** resources (supporting). **Kazuo Horikoshi:** resources (supporting). **Isao Nishiumi:** resources (supporting), writing – review and editing (supporting). **Kazuto Kawakami:** resources (equal), writing – review and editing (supporting). **Yoshio Tsuda:** conceptualization (equal), investigation (supporting), methodology (supporting), supervision (equal), writing – original draft (equal), writing – review and editing (equal). **Koichi Murata:** conceptualization (supporting), funding acquisition (supporting), writing – review and editing (supporting). **Yukita Sato:** conceptualization (lead), funding acquisition (lead), project administration (lead), resources (equal), supervision (lead), validation (equal), visualization (equal), writing – original draft (equal), writing – review and editing (lead).

## Conflicts of Interest

The authors declare no conflicts of interest.

## Supporting information


Data S1.

## Data Availability

The data that support the findings of this study are available in the Supporting Information S1 of this article, and the obtained lineages were deposited to MalAvi and GenBank (Accession numbers LC765283‐LC765287).
