# Supplementary material for: Prevalence and Transmission Cycle of Avian Pathogens in the Isolated Oceanic Islands of Japan
Source: Ecol Evol. 2024 Dec 23;14(12):e70737. doi: 10.1002/ece3.70737 (PMC11666991; doi:10.1002/ece3.70737)
Supplement: Supplementary file 1 — Data S1. [file ECE3-14-e70737-s001.docx]

**Supplementary Information for:**

**Prevalence and transmission cycle of avian pathogens in the Bonin Islands in Japan**

Mizue Inumaru, Rui Kimura, Naoko Suzuki, Hajime Suzuki, Kazuo Horikoshi, Isao Nishiumi, Kazuto Kawakami, Yoshio Tsuda, Koichi Murata, Yukita Sato

**Table of Contents:**

| **Table A1.** Birds sampled in this study, with PCR results of haemosporidian detection | 1-4 |
| --- | --- |
| **Table A2.** Detailed PCR results of resident birds sampled in this study per island | p. 5-6 |
| **Figure A1.** Bayesian phylogenetic analysis of cyt*b* gene lineages (475 bp) of avian haemosporidian parasites, rooted with *Theileria annulata* | p. 7 |
| **Table A3.** Details on microscopy results of smears obtained from birds rescued or captured in Chichijima and Hahajima group, with comparisons to PCR results | p. 8-9 |
| **Figure A2**. Haemosporidian parasites from resident birds of Chichijima | p. 10-11 |
| **Descriptions of parasites found in this study** | p. 12-14 |
| **Figure A3**. Histopathology of a cutaneous lesion from the leg of a warbling white-eye | p. 15 |
| **References** | p. 16 |

**Table A1**. Birds sampled in this study, with PCR results of haemosporidian detection.

| status |  | species | scientific name | Chichijima group | | |  | Hahajima group | | |  | total | | |  | lineages^e^ (number) |
| --- | --- | --- | --- | --- | --- | --- | --- | --- | --- | --- | --- | --- | --- | --- | --- | --- |
|  |  |  |  | sampled | infected | prevalence |  | sampled | infected | prevalence |  | sampled | infected | prevalence |  |  |
| resident |  | Eastern buzzard | *Buteo japonicus toyoshimai*^a^ | 1 | 0 | 0 |  |  |  |  |  | 1 | 0 | 0 |  |  |
|  |  | Black wood pigeon | *Columba janthina nitens*^a^ | 14 | 0 | 0 |  | 1 | 0 | 0 |  | 15 | 0 | 0 |  |  |
|  |  | Brown-eared bulbul | *Hypsipetes amaurotis squamiceps* ^a^ | 11 | 10 | 90.91 |  | 7 | 4 | 57.14 |  | 18 | 14 | 77.78 |  | pGRW04 (1), pGRW06 (10), pCXPIP12 (3) |
|  |  | Japanese bush warbler | *Horornis diphone diphone*^a^ | 14 | 2 | 14.29 |  | 5 | 3 | 0 |  | 19 | 5 | 26.32 |  | pGRW04 (1), pGRW06 (4) |
|  |  | Warbling white-eye | *Zosterops japonicus stejnegeri* ╳  *Z. j. alani*^b^ | 140 | 99^d^ | 70.71 |  | 49 | 37 | 75.51 |  | 189 | 136 | 71.96 |  | pGRW04 (2), pGRW06 (129), pCXPIP12 (1), pMONTRI01 (2), hZOSJAP02^f^ (29) |
|  |  | White's thrush | *Zoothera aurea* | 60 | 22 | 36.67 |  | 7 | 7^d^ | 100 |  | 67 | 29 | 43.28 |  | pGRW06 (19), hZOOLUN01 (4), lZOOAUR01^f^ (6), lZOOAUR02^f^ (1), lZOOAUR03^f^ (1), *Leucocytozoon* sp. [co-infected] (2) |
|  |  | Blue rock thrush | *Monticola solitarius* | 50 | 16 | 32.00 |  | 1 | 0 | 0.00 |  | 51 | 16 | 31.37 |  | pGRW04 (8), pGRW06 (8) |
|  |  | Bonin greenfinch | *Chloris kittlitzi*^a^ |  |  |  |  | 20 | 4 | 20.00 |  | 20 | 4 | 20.00 |  | pGRW06 (4) |
|  |  |  | sub-total | 290 | 149 | 51.38 |  | 90 | 55 | 61.11 |  | 380 | 204 | 53.68 |  |  |
|  |  |  |  |  |  |  |  |  |  |  |  |  |  |  |  |  |
| migrant | migrant breeder | Tristram's storm-petrel | *Hydrobates tristrami* | 1 | 0 | 0 |  |  |  |  |  | 1 | 0 | 0 |  |  |
|  |  | Bonin petrel | *Pterodroma hypoleuca* | 30^c^ | 0 | 0 |  | 1 | 0 | 0 |  | 31 | 0 | 0 |  |  |
|  |  | Wedge-tailed shearwater | *Ardenna pacifica* | 38 | 0 | 0 |  |  |  |  |  | 38 | 0 | 0 |  |  |
|  |  | Bannerman's shearwater | *Puffinus bannermani*^a^ | 5 | 0 | 0 |  |  |  |  |  | 5 | 0 | 0 |  |  |
|  |  | Bulwer's petrel | *Bulweria bulwerii* | 9 | 0 | 0 |  |  |  |  |  | 9 | 0 | 0 |  |  |
|  |  | Brown booby | *Sula leucogaster* | 1 | 0 | 0 |  | 1 | 0 | 0 |  | 2 | 0 | 0 |  |  |
|  |  | Brown noddy | *Anous stolidus* | 1 | 0 | 0 |  |  |  |  |  | 1 | 0 | 0 |  |  |
|  |  | Sooty tern | *Onychoprion fuscatus* | 3 | 0 | 0 |  |  |  |  |  | 3 | 0 | 0 |  |  |
|  | winter visitor | Northern pintail | *Anas acuta* | 1 | 0 | 0 |  |  |  |  |  | 1 | 0 | 0 |  |  |
|  |  | Matsudaira's storm petrel | *Hydrobates matsudairae* |  |  |  |  | 1 | 0 | 0 |  | 1 | 0 | 0 |  |  |
|  |  | Medium egret | *Ardea intermedia* | 2 | 0 | 0 |  |  |  |  |  | 2 | 0 | 0 |  |  |
|  |  | Common moorhen | *Gallinula chloropus* | 3 | 1 | 33.33 |  |  |  |  |  | 3 | 1 | 33.33 |  | *Plasmodium* sp. [co-infected] (1) |
|  |  | Eurasian coot | *Fulica atra* | 1 | 1 | 100 |  |  |  |  |  | 1 | 1 | 100 |  | pSW2 (1) |
|  |  | Pacific golden plover | *Pluvialis fulva* | 23 | 0 | 0 |  |  |  |  |  | 23 | 0 | 0 |  |  |
|  |  | Ruddy turnstone | *Arenaria interpres* | 4 | 0 | 0 |  |  |  |  |  | 4 | 0 | 0 |  |  |
|  | passage visitor | Short-tailed shearwater | *Ardenna tenuirostris* | 2 | 0 | 0 |  |  |  |  |  | 2 | 0 | 0 |  |  |
|  |  | Black-crowned night-heron | *Nycticorax nycticorax* | 1 | 0 | 0 |  |  |  |  |  | 1 | 0 | 0 |  |  |
|  |  | Barn swallow | *Hirundo rustica* | 1 | 0 | 0 |  |  |  |  |  | 1 | 0 | 0 |  |  |
|  | irregular or | Eurasian teal | *Anas crecca* | 1 | 1 | 100 |  |  |  |  |  | 1 | 1 | 100 |  | lANACRE04^f^ (1) |
|  | accidental visitor | Leach's storm-petrel | *Hydrobates leucorhous* | 1 | 0 | 0 |  |  |  |  |  | 1 | 0 | 0 |  |  |
|  |  | Great egret | *Ardea alba* | 1 | 0 | 0 |  |  |  |  |  | 1 | 0 | 0 |  |  |
|  |  | Great frigatebird | *Fregata minor* |  |  |  |  | 1 | 0 | 0 |  | 1 | 0 | 0 |  |  |
|  |  | Black-winged stilt | *Himantopus himantopus* | 2 | 1 | 50.00 |  |  |  |  |  | 2 | 1 | 50.00 |  | pACCBAD01 (1) |
|  |  | Little ringed plover | *Charadrius dubius* | 1 | 0 | 0 |  |  |  |  |  | 1 | 0 | 0 |  |  |
|  |  | Common snipe | *Gallinago gallinago* | 1 | 0 | 0 |  |  |  |  |  | 1 | 0 | 0 |  |  |
|  |  | Oriental pratincole | *Glareola maldivarum* | 2 | 0 | 0 |  |  |  |  |  | 2 | 0 | 0 |  |  |
|  |  | Whiskered tern | *Chlidonias hybrida* | 1 | 0 | 0 |  |  |  |  |  | 1 | 0 | 0 |  |  |
|  |  | White-winged tern | *Chlidonias leucopterus* | 1 | 0 | 0 |  |  |  |  |  | 1 | 0 | 0 |  |  |
|  |  | Oriental turtle dove | *Streptopelia orientalis* | 1 | 0 | 0 |  |  |  |  |  | 1 | 0 | 0 |  |  |
|  |  | Hawfinch | *Coccothraustes coccothraustes* | 1 | 1 | 100 |  |  |  |  |  | 1 | 1 | 100 |  | pGRW06 (1) |
|  |  | Red crossbill | *Loxia curvirostra* | 1 | 0 | 0 |  |  |  |  |  | 1 | 0 | 0 |  |  |
|  |  |  | sub-total | 140 | 5 | 3.57 |  | 4 | 0 | 0 |  | 144 | 5 | 3.47 |  |  |
|  |  |  |  |  |  |  |  |  |  |  |  |  |  |  |  |  |
|  |  |  | total | 430 | 154 | 35.81 |  | 94 | 55 | 58.51 |  | 524 | 209 | 39.89 |  |  |

^a^ Endemic species/subspecies

^b^ Introduced species

^c^ One Bonin petrel was rescued on the passenger ship Ogasawara-maru

^d^ Co-infected individuals were detected. 26 Warbling white-eyes: pGRW06 x hZOSJAP02; 1 warbling white-eye: pMONTRI01 x hZOSJAP02; 1 White's thrush: pGRW06 x lZOOAUR01; 1 White's thrush: pGRW06 x lZOOAUR03, 1 White's thrush: hZOOLUN01 x lZOOAUR01.

^e^ Lineage names are given according to MalAvi database

^f^ Lineages detected for the first time

**Table A2.** Detailed PCR results of resident birds sampled in this study per island.

|  | *But.jap*^a^ |  | *Col. jan* |  | *Hyp. amaurotis* | |  | *Hor. diphone* | |  | *Zos. japonicus* | |  | *Zoo. aurea* | |  | *M. solitarius* | |  | *Chl. kittlitzi* | |
| --- | --- | --- | --- | --- | --- | --- | --- | --- | --- | --- | --- | --- | --- | --- | --- | --- | --- | --- | --- | --- | --- |
| island | pos/no.^b^ |  | pos/no. |  | pos/no. | lineages^c^ |  | pos/no. | lineages |  | pos/no. | lineages |  | pos/no. | lineages |  | pos/no. | lineages |  | pos/no. | lineages |
| Chichijima | 0/1 |  | 0/14 |  | 10/11 | pGRW04 (1) |  | 2/13 | pGRW04 (1) |  | 99/140 | pGRW04 (2) |  | 22/60 | pGRW06 (16) |  | 16/50 | pGRW04 (8) |  |  |  |
|  |  |  |  |  |  | pGRW06 (7) |  |  | pGRW06 (1) |  |  | pGRW06 (93) |  |  | lZOOLUN01 (2) |  |  | pGRW06 (8) |  |  |  |
|  |  |  |  |  |  | pCXPIP12 (2) |  |  |  |  |  | pCXPIP12 (1) |  |  | lZOOAUR01 (1) |  |  |  |  |  |  |
|  |  |  |  |  |  |  |  |  |  |  |  | pMONTRI01 (2) |  |  | lZOOAUR02 (1) |  |  |  |  |  |  |
|  |  |  |  |  |  |  |  |  |  |  |  | hZOSJAP02 (16) |  |  | l [co-inefcted] (2) |  |  |  |  |  |  |
| Anijima |  |  |  |  |  |  |  | 0/1 |  |  |  |  |  |  |  |  |  |  |  |  |  |
| Hahajima |  |  | 0/1 |  | 1/3 | pCXPIP12 (1) |  |  |  |  |  |  |  |  |  |  |  |  |  |  |  |
| Mukohjima |  |  |  |  | 0/1 |  |  | 2/3 | pGRW06 (2) |  | 16/22 | pGRW06 (16) |  | 5/5 | pGRW06 (2) |  |  |  |  | 0/5 |  |
|  |  |  |  |  |  |  |  |  |  |  |  | hZOSJAP02 (9) |  |  | hZOOLUN01 (2) |  |  |  |  |  |  |
|  |  |  |  |  |  |  |  |  |  |  |  |  |  |  | lZOOAUR01 (4) |  |  |  |  |  |  |
|  |  |  |  |  |  |  |  |  |  |  |  |  |  |  | lZOOAUR03 (1) |  |  |  |  |  |  |
| Meijima |  |  |  |  |  |  |  | 0/1 |  |  | 5/8 | pGRW06 (4) |  |  |  |  |  |  |  | 0/2 |  |
|  |  |  |  |  |  |  |  |  |  |  |  | hZOSJAP02 (2) |  |  |  |  |  |  |  |  |  |
| Imotojima |  |  |  |  |  |  |  |  |  |  | 1/1 | pGRW06 (1) |  |  |  |  | 0/1 |  |  | 0/2 |  |
|  |  |  |  |  |  |  |  |  |  |  |  | hZOSJAP02 (1) |  |  |  |  |  |  |  |  |  |
| Anejima |  |  |  |  | 3/3 | pGRW06 (3) |  | 1/1 | pGRW06 (1) |  | 15/18 | pGRW06 (15) |  | 2/2 | pGRW06 (1) |  |  |  |  | 4/11 | pGRW06 (4) |
|  |  |  |  |  |  |  |  |  |  |  |  | hZOSJAP02 (1) |  |  | lZOOAUR01 (1) |  |  |  |  |  |  |
| total | 0/1 |  | 0/15 |  | 14/18 |  |  | 5/19 |  |  | 136/189 |  |  | 29/67 |  |  | 16/51 |  |  | 4/20 |  |

^a^ Species names are abbreviated. Full scientific names are as follows: *Buteo japonicus*, *Columba janthina*, *Hypsipetes amaurotis*, *Horornis diphone*, *Zosterops japonicus*, *Zoothera aurea*, *Monticola solatarius*, *Chloris kittlitzi*

^b^ pos/no.: positive samples/negative samples

^c^ Lineage names are given according to MalAvi database. Parentheses following the lineage names show the number per lineage.

**
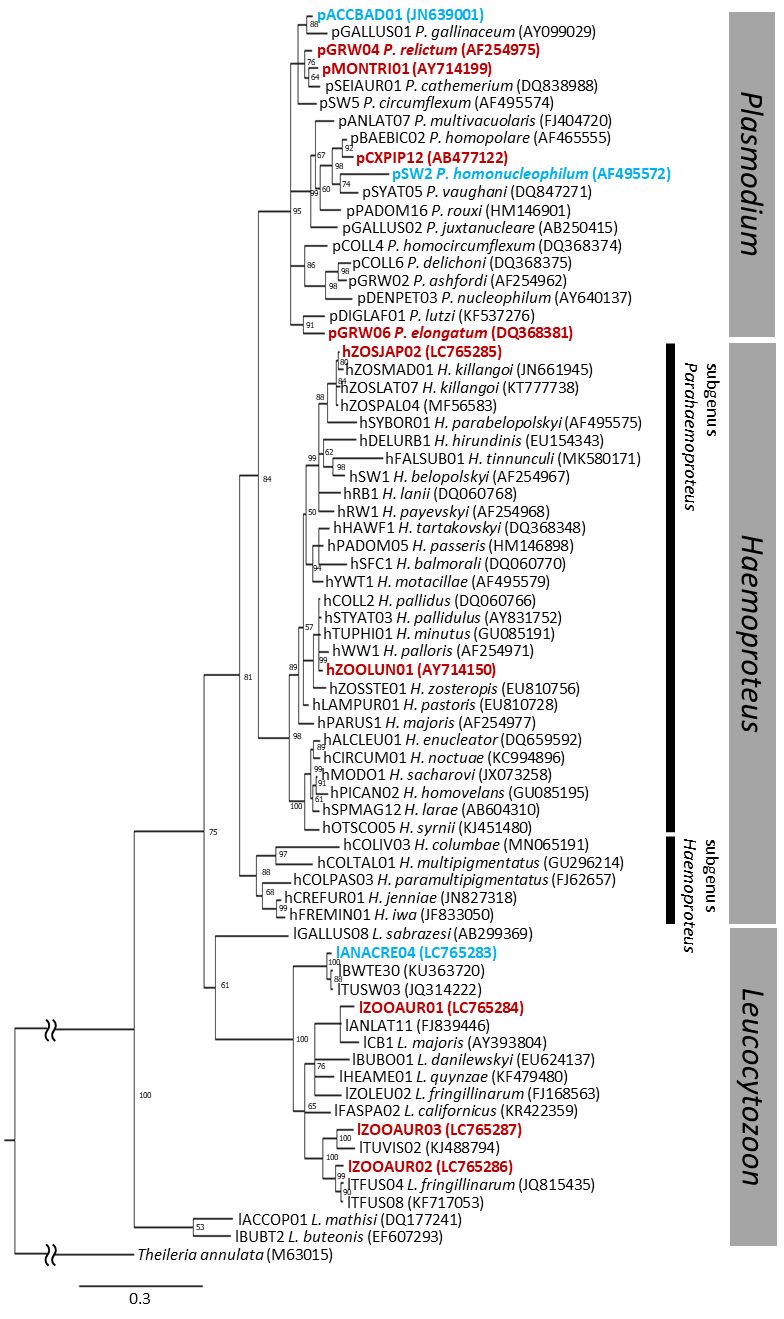
Figure A1**. Bayesian phylogenetic analysis of cyt*b* gene lineages (475 bp) of avian haemosporidian parasites, rooted with *Theileria annulata*. Only posterior clade probabilities of >0.60 were indicated. Branch lengths are proportional to the amount of change according to the applied substitution model. Lineages from resident birds are shown in red letters, and lineages detected from only migratory birds are shown in blue letters.

**Table A3**. Details on microscopy results of smears obtained from birds rescued or captured in Chichijima and Hahajima group, with comparisons to PCR results.

| host species | smears | positive^a^ | microscopy results / PCR results (number) |
| --- | --- | --- | --- |
| *Hypsipetes amaurotis squamiceps* | 7 | 4 (5) | *P. elongatum* / pGRW06 (2)  *P. relictum* / pGRW04 (1)  *Plasmodium* sp. / pCXPIP12 (1) |
| *Horornis diphone diphone* | 7 | 1 (3) | *P. relictum* / pGRW06 (1) |
| *Zosterops japonicus* | 65 | 59 (60) | *P. elongatum* / pGRW06 (29)  *P. elongatum* x *P. relictum* / pGRW06 (2)  *P. elongatum* x *H. killangoi* / pGRW06 x hZOSJAP02 (19)  *P. elongatum* x *H. killangoi* / pGRW06 (2)  *P. relictum* / pGRW04 (1)  *Plasmodium* sp. / pGRW06 (6) |
| *Zoothera aurea* | 5 | 5 | *P. elongatum* / pGRW06 (2)  *H. pallidus* x *L. minutus* / hZOOLUN01 x ZOOAUR01 (2)  *L. minutus* / lZOOAUR01 (1) |
| *Monticola solitarius* | 4 | 2 | *P. relictum* / pGRW04 (2) |
| *Chloris kittlitzi* | 10 | 2 | *P. elongatum* / pGRW06 (2) |
| *Pterodroma hypoleuca* | 1 | 0 | - |
| *Sula leucogaster* | 1 | 0 | - |
| Total | 100 | 71 (77) |  |

^a^ Number in parentheses are the number of PCR-positive individuals. If no parentheses are shown, the number of PCR-positive individuals is equal to the number of smear-positive individuals. Note that the lineage pGRW06 was detected by PCR from all 6 individuals which were PCR-positive and smear-negative.

**Figure A2**. Haemosporidian parasites from resident birds of Chichijima. *Plasmodium elongatum* from mist-netted warbling white-eyes (*Zosterops japonicus*) (a-c) and an Ogasawara greenfinch (*Chloris kittlitzi*); *Plasmodium relictum* from a rescued brown-eared bulbul (*Hypsipetes amaurotis*) (e) and deceased blue rock thrush (*Monticola solitarius*) (f, g); *Plasmodium* sp. from a mist-netted brown-eared bulbul (h); *Haemoproteus zosteropis* from mist-netted warbling white-eyes (i-n); *Haemoproteus pallidus* from White’s thrush (*Zoothera aurea*) (o-r); *Leucocytozoon majoris* from White’s thrush (s, t). Young gametocyte (i), microgametocyes (a, b, h, m-q, s), macrogametocytes (c-f, j-l, q, t), macrogamete (r), microgamete (r), and erythrocytic meront (g). Stained with Hemacolor®. Scale-bar: 10 µm.

*
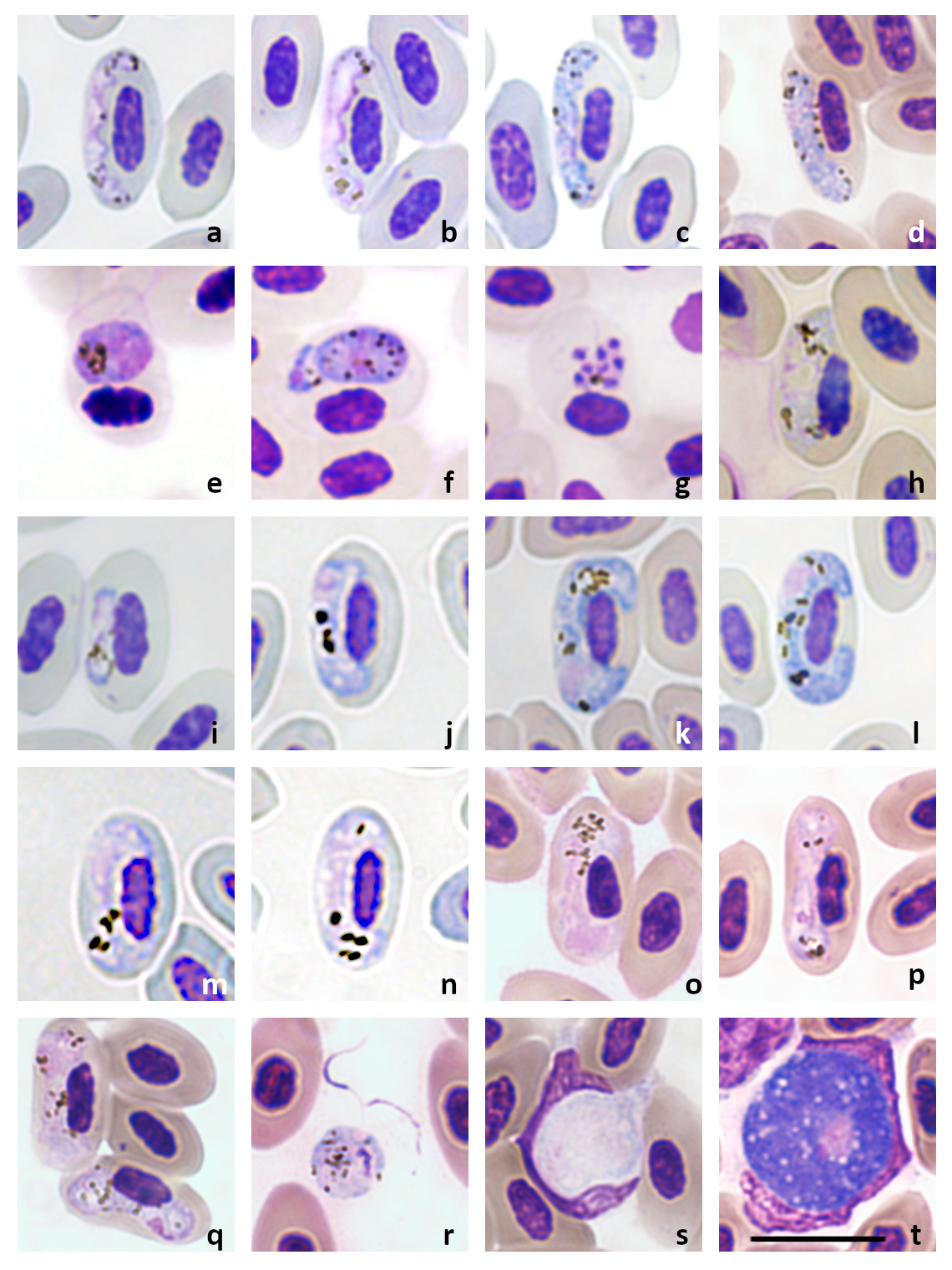
*

**Descriptions of parasites found in this study:**

***Haemoproteus* (*Parahaemoproteus*) *killangoi* Bennett and Peirce, 1981**

*Host*: The lineage hZOSJAP02 was recorded in the warbling white-eye (*Zosterops japonicus*).

*Locality*: Chichijima, Bonin Islands, Tokyo, Japan.

*Representative DNA sequences*: Mitochondrial cyt*b* lineage hZOSJAP02 (478bp, GenBank accession number: LC765285).

*Representative blood films*: Voucher specimens (MPM Coll. No. 25289) were deposited in the Meguro Parasitological Museum, Tokyo, Japan.

*Site of infection*: Mature erythrocytes; no other data.

*Young gametocytes* (fig. A2 I) adhere to the erythrocyte nucleus. The outline is slightly ameboid.

*Macrogametocytes* (fig. A2 j-l) grow around the erythrocyte nucleus, enclosing them with ends. However, gametocytes do not complete encircle the erythrocyte nucleus. Growing gametocytes do not displace erythrocyte nucleus, but mature gametocytes may occasionally displace erythrocyte nucleus laterally. Gametocytes form dumbbell-like shapes during growth. Fully grown gametocytes lose the ameboid outline and dumbbell-like shape, displaying an even outline appressed to the nucleus and envelope of the erythrocyte (fig. A2 k, l). Parasite nucleus is compact and subterminal in position (fig. A2 j-l). Round and medium-sized (0.5-1.0 µm) (fig. A2 k), or large rod-like pigment granules (fig. A2 j-l) are present; 7-11 (average 9.2 ± 1.3) in number.

*Microgametocytes* (fig. A2 m, n). The general configuration and other features are as for macrogametocytes, with usual haemosporidian sexual dimorphic characters (Valkiūnas 2005).

*Remarks*: Mixed infections of *Haemoproteus killangoi* and *H. zosteropis* have been recorded often. The former can be distinguished from the latter based on the smaller number of pigment granules and ameboid appearance of young to growing gametocytes. Additionally, macrogametocytes of *H. killangoi* are predominantly nucleophilic, while *H. zosteropis* tend to be closely appressed to the erythrocyte envelope.

***Haemoproteus* (*Parahaemoproteus*) *pallidus* Valkiūnas and Iezhova, 1991**

*Host*: The lineage hZOOLUN01 was recorded in the White’s thrush (*Zoothera aurea*).

*Locality*: Mukohjima, Bonin Islands, Tokyo, Japan.

*Representative DNA sequences*: Mitochondrial cyt*b* lineage hZOOLUN01 (478bp, GenBank accession number: AY714150).

*Representative blood films*: Voucher specimens (MPM Coll. No. 25290) were deposited in the Meguro Parasitological Museum, Tokyo, Japan.

*Site of infection*: Mature erythrocytes; no other data.

Macrogametocytes (fig. A2 q) closely appress to the erythrocyte nucleus, enclosing the nucleus with their ends but do not completely. Gametocytes do not touch the envelope of erythrocytes along the entire margin, forming an irregular ‘cleft’ between the parasite and erythrocyte envelope. Fully grown gametocytes slightly displace the erythrocyte nucleus laterally and to not fill the poles of the erythrocyte. The outline may be even or ameboid. Parasites stain pale and are similar in appearance to microgametocytes. Parasite nuclei are subterminal or occasionally terminal, and usually elongated. Pigment granules are generally round and small (<0.5 µm) to medium (0.5 to 1.0 µm) in size. These granules are scattered throughout the cytoplasm.

Microgametocytes (fig. A2 o-q) The general configuration and other features are as for macrogametocytes, with usual haemosporidian sexual dimorphic characters (Valkiūnas 2005). Due to the pale staining of macrogametocytes, micro- and macrogametocytes are poorly distinguishable. Parasite nuclei are diffuse (fig. A2 o) but may also be more compact (fig. A2 p, q).

***Leucocytozoon* (*Leucocytozoon*) *majoris* (Laveran, 1902)**

*Host*: The lineage lZOOAUR01 was recorded in the White’s thrush (*Zoothera aurea*).

*Locality*: Mukohjima, Bonin Islands, Tokyo, Japan

*Representative DNA sequences*: Mitochondrial cyt*b* lineage lZOOAUR01 (478bp, GenBank accession number: LC765284).

*Representative blood films*: Voucher specimens (MPM Coll. No. 25291) were deposited in the Meguro Parasitological Museum, Tokyo, Japan.

*Site of infection*: Mature host cells; no other data.

Macrogametocytes (fig. A2 t) develop in round host cells. The cytoplasm often contains numerous small vacuoles. Parasite nuclei are variable in both form and position. Nucleoli are prominent and easily distinguishable. The host cell nucleus is pushed aside and deformed, creating a band which lines peripherally and extends more than 1/2 of the gametocyte circumference. The cytoplasm of the host cell is mostly replaced by the gametocyte and lies around the periphery of the gametocyte.

Microgametocytes (fig. A2 s) A2 o-q) The general configuration and other features are as for macrogametocytes, with usual haemosporidian sexual dimorphic characters (Valkiūnas 2005).

**Figure A3**. Histopathology of a cutaneous lesion from the leg of a warbling white-eye. Magnification x200 (a) and x600 (b). Haematoxylin and eosin staining. Scale-bar: 60 µm (a) and 20 µm (b).


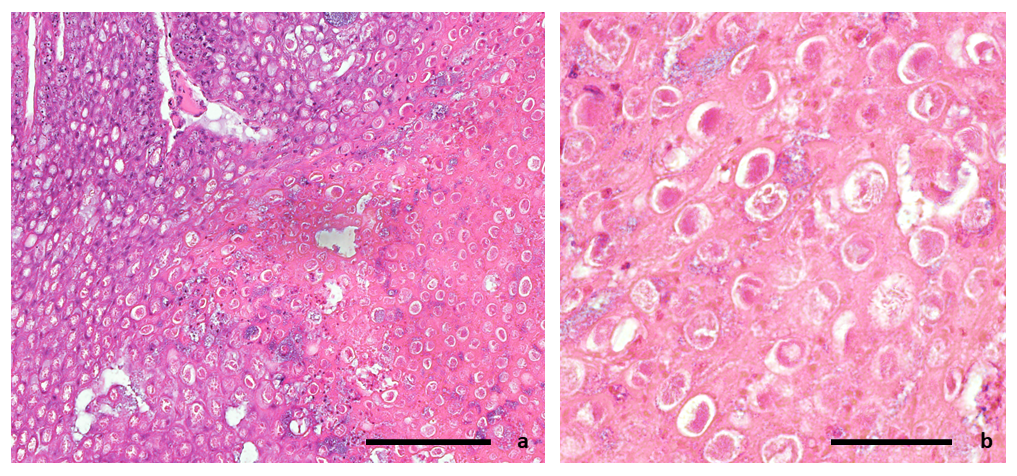


Hyperplasia of the epithelium with vacuolar degeneration and intracytoplasmic inclusion bodies (Bollinger bodies) was observed, confirming infection of APV. However, note that abnormal staining and postmortem bacterial growth were also observed. The bird was frozen before necropsy and samples were obtained after defrosting. Freezing is generally known to create artifacts and histopathological observations may be obscured (Rae 2003, Steu et al. 2008, Taqi et al. 2018). The unusual staining and unclear shape of the Bollinger bodies may be effects of freezing. Additionally, the observed postmortem bacterial growth is thought to be due to a delay in freezing after the bird’s death. Although observations were not made in the best condition, it was still possible to identify APV infection.

**Reference:**

Rae, M. A. 2003. Practical avian necropsy. - Semin. Avian Exot. Pet Med. 12: 62–70.

Steu, S., Baucamp, M., Von Dach, G., Bawohl, M., Dettwiler, S., Storz, M., Moch, H. and Schraml, P. 2008. A procedure for tissue freezing and processing applicable to both intra-operative frozen section diagnosis and tissue banking in surgical pathology. - Virchows Arch 452: 305–312.

Taqi, S. A., Sami, S. A., Sami, L. B. and Zaki, S. A. 2018. A review of artifacts in histopathology. - J. Oral Maxillofac. Pathol. 22: 279.

Valkiūnas, G. 2005. Avian malaria parasites and other haemosporidia. - CRC Press.
